# 4-Hydroxy-3-Methyl-2-Alkenylquinoline Disrupts Temporospatial Control of Flagellar Assembly and Induces Transcriptomic Remodeling in *Bacillus subtilis* Consistent with a Short-Circuiting of the Spo0A Phosphorelay

**DOI:** 10.3390/molecules31183226

**Published:** 2026-09-12

**Authors:** McKinley D. Williams, Leif Smith

**Affiliations:** 1 Department of Biology, Texas A&M University, College Station, TX 77843, USA; mcdwilliams@pvamu.edu; 2 Department of Biology, Prairie View A&M University, Prairie View, TX 77446, USA; 3 Antimicrobial Division, Sano Chemicals Inc., Bryan, TX 77808, USA

**Keywords:** anti-sporulation, anti-biofilm, antimicrobial, Spo0A, *Bacillus subtilis*, flagella, *Burkholderia*, alkaloid, quinolone

## Abstract

4-hydroxy-3-methyl-2-alkenylquinolines (HMAQs) belong to an emerging sub-class of naturally synthesized alkaloids with several prominent biological properties. HMAQ-7 is a novel variant recently identified in *Burkholderia contaminans* MS14 which was previously shown to impede normal cellular behaviors in *Bacillus subtilis*, including multiple colonization phenotypes such as motility, pellicle formation, and sporulation. While the list of biological and chemical characteristics among HMAQ’s and related molecules is extensive, the mechanistic basis of these responses has been far more elusive. Using a combination of RNA-seq and bioinformatics, we have determined that several of the physiological defects induced by HMAQ-7 are strongly correlated with alterations in systems related to phospho-transduction. Specifically, HMAQ-7 augments gene expression in elements associated with late stationary phase growth, including those involved in phosphate assimilation and regulation of the spo0A phosphorelay. We also note that HMAQ-7 promotes dysregulated transcription of flagellar assembly genes which would normally be downregulated during late stationary phase. This study constitutes one of the first extensive investigations into an HMAQ’s mode of action, providing vital insight into the ecological dynamics between *B. contaminans* and *B. subtilis.*

## 1. Introduction

**Figure 1:**
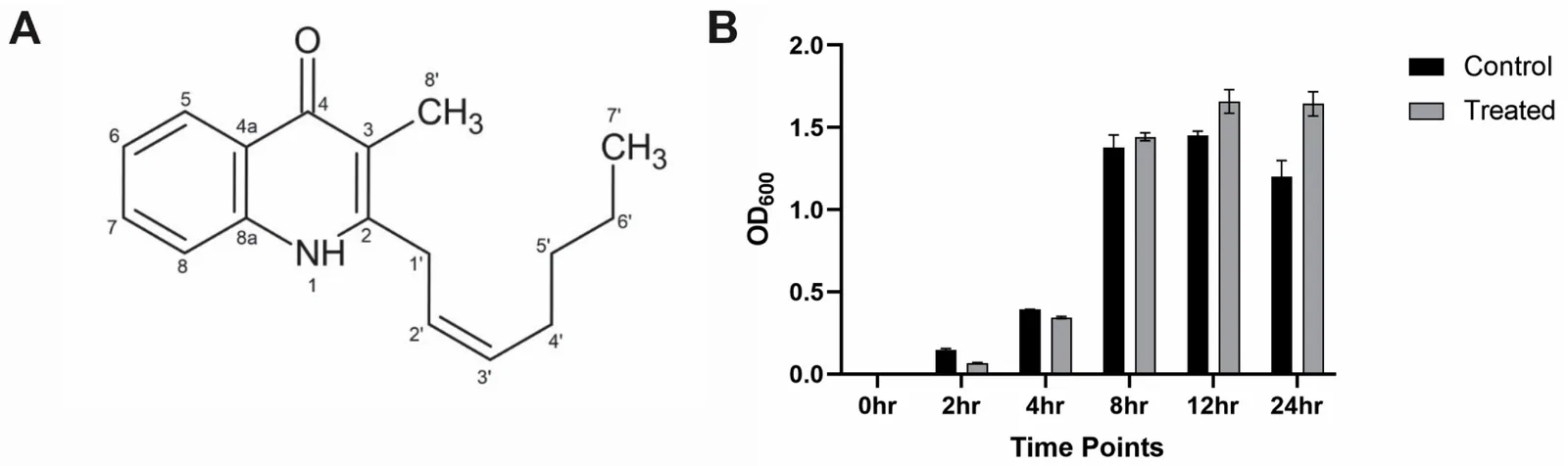
HMAQ-7 is a naturally occurring *Burkholderia* quinoline that augments *B. subtilis* culture growth during the stationary phase. (**A**) Covalent structure of HMAQ-7. (**B**) OD_600_ measurements of *B. subtilis* culture exposed to HMAQ-7 over the course of 24 h.

Soil and aquatic bacteria have long been considered key sources for the identification of novel antimicrobial metabolites. Competition amongst the myriad clades and serotypes of these terraqueous species has led them to cultivate a diverse arsenal of molecular weaponry suitable for interspecies conflict. *Burkholderia* species are well established producers of a wide assortment of antibacterial, antifungal, antiparasitic, and anticancer compounds with novel mechanisms of action [1,2,3,4,5,6,7,8,9,10]. In particular, *B. contaminans* MS14 produces the antifungal occidiofungin [11,12,13], the siderophore ornibactin [14], and the quinolone 4-hydroxy-3-methyl-2-alkenylquinoline-C7 (HMAQ-7) (Figure 1A) [15].

The HMAQs belong to a subdivision of naturally derived alkaloids commonly produced by *Burkholderia* [16]. They have been the subject of multiple investigations into the structure–function dynamics of natural compounds, with these assessments often revealing several unique biological properties [15,17,18]. Like many alkaloids, the HMAQs are small heterocyclic compounds of rudimentary composition, consisting of many varieties of fused bicyclic rings which are alkylated at the C2 carbon. Unique among the HMAQ’s, and a point of great interest, is the characteristic methylation of their C3 carbon, an addition critical to their function [6,18,19,20]. HMAQs possess structural affinity with known quorum sensing (QS) agents, 2-heptyl-alkyl-quinolones (HAQs), which are synthesized by *Pseudomonas aeruginosa* and known to promote the expression of several virulence factors, including biofilm production, siderophore release, and autoaggregation [21,22,23,24]. By contrast, HMAQ’s actively inhibit several of these virulent multi-cellular behaviors, with many also possessing bacteriostatic, cidal, and cytotoxic properties [15,18,25]. Furthermore, unlike HAQ’s and similar QS signaling molecules which are autoinducers, HMAQ’s do not promote their own biosynthesis and are only able to interact indirectly in QS-mediated transcription via modulating the expression levels of N-acyl-homoserine lactone variants in bacterial congeners [26].

A great deal has been established about HMAQ’s and related heterocyclic compounds, particularly with respect to their chemical properties, including structural diversity, the significance of their functional groups, and how the degrees of unsaturation within their alkyl chains affect signaling capabilities [18,27,28,29]. What is far less established is the precise mode(s) of action (MoA) that underpin most biological responses associated with HMAQ exposure. The current literature provides neither experimental nor theoretical elucidations into the mechanics of HMAQ’s phenotypic inductions. This paucity in robust MoA research is striking given the various responses HMAQs have shown to induce across diverse bacterial species, particularly in their hindrance of bacterial growth. The potential for HMAQs to inhibit microbial growth or disrupt microbial virulence has led to an increased interest in exploring the biophysiological aspects behind HMAQ’s activity [15,17,30].

Our group’s earlier investigation into HMAQ-7 demonstrated that the compound did not exert growth inhibition against most bacterial and fungal species that were tested. However, at low concentrations, the compound was able to disrupt complex multi-species biofilms, suppress motility, and abrogate colonization phenotypes associated with transitional and stationary phases of culture growth, including pellicle formation and sporulation in *Bacillus subtilis* [15]. We became interested in whether HMAQ-7 exposure was tied to any corresponding cellular adaptations or alterations in viability. Consistent with these earlier observations, *B. subtilis* exposure to HMAQ-7 did not appear to affect a noticeable change in cell growth during either the lag or logarithmic phases. Rather, significant differences were observed during stationary phase, wherein treated cultures outpaced controls in terms of their relative cell density and did not experience a decline in OD_600_ at 24 h (Figure 1B).

Typically, decreases in cellular density during late stationary phase are characteristic of *Bacillus* and *Clostridium* species undergoing autolysis, a process closely linked to nutrient depletion and later stages of sporulation [31,32,33]. The apparent absence of this phenomenon among treated cell cultures suggested that HMAQ-7 was likely interfering with several additional late stationary-phase adaptations that led to the observed higher cell densities. These observations led us to employ RNA-seq to better establish the transcriptomic profile of *B. subtilis* under HMAQ-7 exposure. In doing so, we endeavored to close the knowledge gap surrounding the mechanistic basis for HMAQ’s anti-colonization phenotypes and provide a foundation for future investigations.

## 2. Results and Discussion

### 2.1. Transcriptomic Profile of Treated B. subtilis

**Figure 2:**
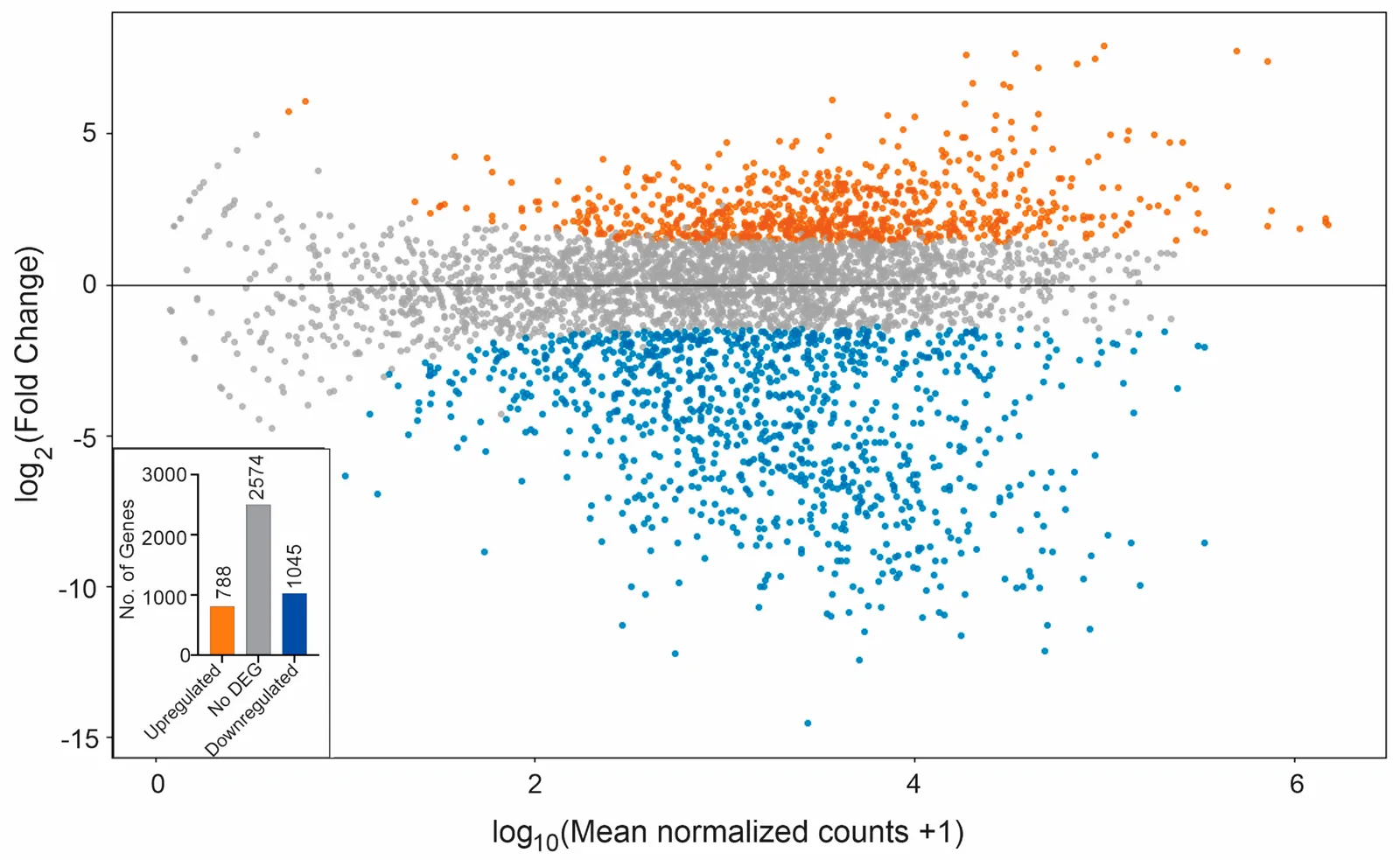
Exposure to HMAQ-7 induces significant changes in transcriptional expression during late stationary phase. MA plot depicts all DEGs (*p*adj < 0.05; |Log_2_FC| ≥ 1.5) and non-DEGs detected in the RNA-seq analysis mapped against their associated log fold changes. The inset provides the corresponding number of genes in each category.

We noted in our previous study that when *B. subtilis* is exposed to a minimum effective concentration (MEC) of 10 µg/mL HMAQ-7 in standing culture, pellicle formation is ablated and sporulation is significantly diminished by 24 h [15]. For RNA-seq culture harvesting, we employed an identical experimental setup, allowing *B. subtilis* to incubate with HMAQ-7 in static culture over the same period just prior to collection. Consistent with our previous results, we observed differences in pellicle formation between treated and control cultures but noted that both cultures achieved equivalent cell densities of ~8 × 10^9^ CFU/mL prior to culture harvesting. Both Principal Component Analysis (PCA) and Pearson’s correlation analysis showed that the control and treated groups had distinct transcriptomic profiles (Figure S1). RNA-seq analysis revealed that *B. subtilis* experiences a sizable alteration in its transcriptome in response to prolonged HMAQ-7 exposure under experimental conditions. Of the 4407 genes detected, 788 (17.90%) were upregulated (*p*adj < 0.05; Log_2_FC ≥ 1.5), 1045 (23.70%) were downregulated (*p*adj < 0.05; Log_2_FC ≤ −1.5), and 2574 (58.40%) were not differentially expressed (*p*adj ≥ 0.05) (Figure 2). Such marked changes in global gene expression are consistent with HMAQ-7 being a type of QS-like signal molecule, behaving as an exogenous transcriptional regulator. Given that this response was observed in a species lacking the requisite biosynthesis genes needed for HMAQ-mediated cross-signaling, HMAQ-7 could be described as a molecular parainducer, similar to autoinducer 2 (AI-2), noted for its unidirectional QS-signaling in *Mycobacterium avium* and *Pseudomonas aeruginosa* [34,35]. Further supporting this view, we observed that among the top 25 genes which were downregulated in *B. subtilis*, nearly all of them were involved in sporulation and under the control of the transcriptional master regulator, Spo0A (Table 1).

### 2.2. GO Term and KEGG Enrichment Analysis

**Figure 3:**
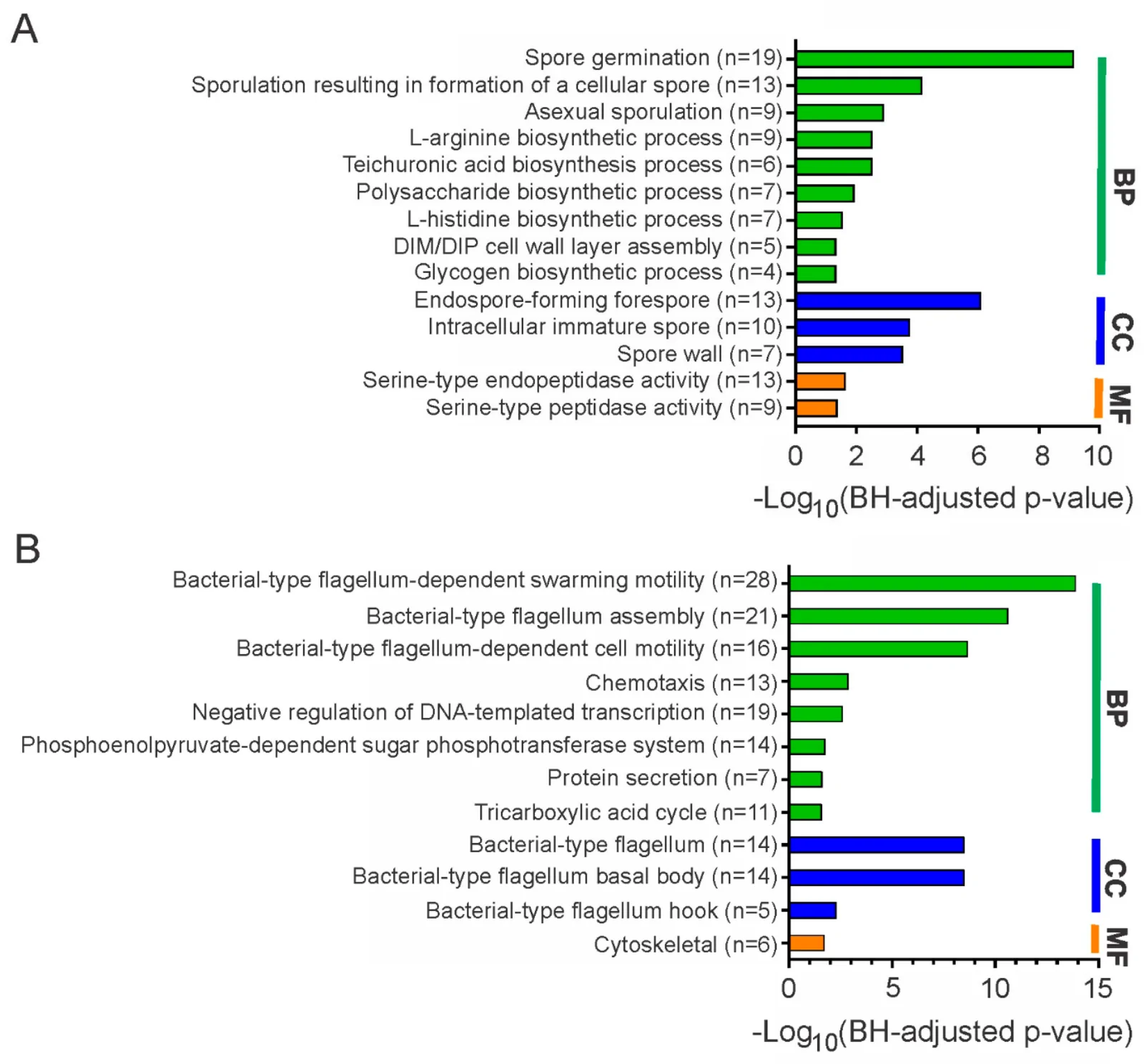
HMAQ-7 promotes the upregulation of genes involved in flagellar assembly and motility while suppressing expression of genes involved in sporulation during late stationary phase in *B. subtilis*. Enrichment analysis identifies a number of GO terms that are either downregulated or upregulated 24 h into HMAQ-7 incubation. (**A**) GO enrichment of downregulated genes. (**B**) GO enrichment of upregulated genes. The “n” next to each pathway denotes the number of differentially expressed genes (*p*adj < 0.05) therein.

Enrichment analyses were performed to better contextualize global changes to the transcriptome and tie these alterations to any relevant biological and/or biochemical pathways. Among all differentially expressed genes (DEGs) that experienced significant downregulation, Gene Ontology (GO) enrichment identified the top 3 categories among “Biological Processes” (BP) as being associated with sporulation (Figure 3A). Concomitantly, enrichment categories among “Cellular Components” (CC) were exclusively tied to sporulation-related elements. The same analysis done with upregulated genes identified the most enriched categories among BPs and CCs as being associated with flagellum synthesis and motility (Figure 3B). Kyoto Encyclopedia of Genes and Genomes (KEGG) pathway analysis revealed comparable results, showing that biosynthesis pathways involved in flagellar assembly and bacterial chemotaxis were the most highly enriched in the *B. subtilis* transcriptome (Figures S1 and S2).

Flagellar assembly is commonly associated with exponential cell growth during the latter stages of log phase rather than in late stationary phase where severe nutrient deprivation would make synthesis far too costly [36,37,38,39]. Considering that our previous findings showed HMAQ-7 inhibiting motility of *B. subtilis* in low-density agars, we found such an increase in transcription of genes related to motility to be highly paradoxical [15]. This decoupling of flagellar assembly from standard nutritional cues within cell culture would suggest that HMAQ-7 is mediating a maladaptive and/or severely dysregulated stress response. Indeed, among the top 25 upregulated genes, several are tied to nutrient uptake (salts, metal ions, carbohydrates), with KEGG enrichment revealing upregulation of inositol phosphorylation and phosphotransferase pathways, consistent with a starvation response (Table 2; Figure S3).

Besides enrichment of phosphotransferase and inositol phosphorylation systems, KEGG analysis also identified sizable enrichment in many elements associated with the two-component system pathways of *B. subtilis*. Using an exploratory *p*adj-value cutoff of 0.1 in tandem with the pathview tool [40], several genes associated with two-component systems (TCS) were mapped to their respective regulatory schemes, revealing that many of these pathways were undergoing modulation, including those for nitrate reduction, protein secretion, and citrate utilization (Figure S4). Of these TCS-associated DEGs, the two most interesting were the downregulated genes *phoB*, which encodes an alkaline phosphatase involved in phosphate assimilation [41,42], and *kinE*, which encodes a regulatory kinase involved in phosphorylation of Spo0A [43,44]. The phosphorylation state of various regulatory proteins is essential for controlling several physiological behaviors within a bacterial cell, including sporulation, motility, biofilm formation, and metabolic adaptations [45,46,47,48,49]. Considering these observations, we came to hypothesize that HMAQ-7 might be achieving its inhibitory phenotypes via interference with signal transduction within *B. subtilis*.

### 2.3. Transcriptional Expression of Spo0A Phosphorelay Components

**Figure 4:**
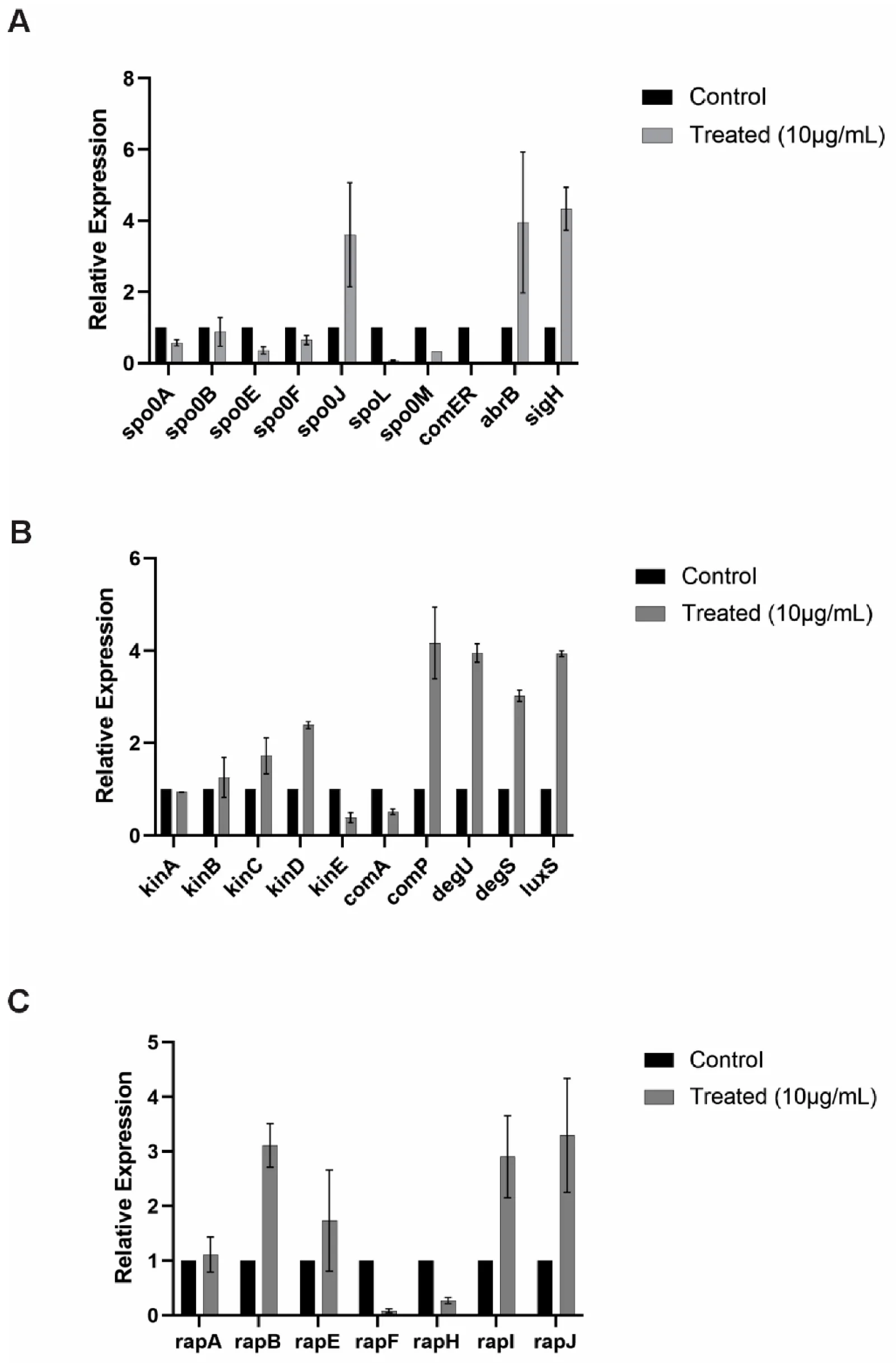
HMAQ-7 modulates the transcription of several regulatory elements in the spo0A phosphorelay circuit. (**A**) Relative transcription levels of elements within the spo0 regulon. (**B**) Relative transcription levels of kinases and competence genes associated with the spo0 regulon and motility in *B. subtilis*. (**C**) Relative transcription levels of phosphatases involved in controlling the phosphorylation state of various Spo0 proteins.

As a model organism, several regulatory pathways and systems in *B. subtilis* have been thoroughly investigated and well characterized, including those related to starvation, motility, sporulation, and biofilm formation [50,51,52,53,54,55,56,57,58]. Given our exploratory findings in pathview and the fact that biofilm formation and sporulation are tightly linked through the transcription master regulator, Spo0A, we thought it best to focus on the phosphorelay system [59,60]. It is well established that *spo0A* transcription is autoregulated, heterogeneous in cell culture, and bistable, with basal-level expression occurring during vegetative growth, and heightened transcription occurring at the start of the transition period [61,62,63,64,65]. We were interested to see whether the decline in sporulation and pellicle formation reflected a defect in a single regulatory component or a broader global downregulation of the associated operons. Normalized transcript counts for each gene were used to evaluate relative expression levels of the major regulatory components. With the exception of *spo0J*, all *spo0* class transcripts experienced downregulation (Figure 4A; Table 3). Additionally, other *spo0A*-adjacent regulators such as sigma factor H and the transition state regulator, *abrB*, experienced significant upregulation while *comER* transcription was entirely ablated.

Both *sigH* and *spo0J* are known to promote transcription of *spo0A* during the latter stages of culture growth [44,66,67]. Reduced cell growth experienced by *B. subtilis* during this period leads to increased phosphorylation of Spo0A, which, being in its active form, promotes sporulation/biofilm formation and serves as a direct inhibitor of abrB. While direct experimental data would be required to support this, the reduced transcription of *spo0A* despite increased transcription of *sigH* and *spo0J* is consistent with a significant break in the phosphorelay circuit. The ablation of *comER* transcription further supports this hypothesis given that this gene is essential for the time-sensitive phosphorylation of the intermediate phosphoryl-carrier, Spo0F, which operates upstream of Spo0A within the circuit [58]. *spo0A* transcription levels are dependent in large part on the availability of Spo0A~P which is modulated under natural conditions via multiple phosphatases and kinases including several varieties of rap proteins and KinA-E [44,68,69,70]. When looking at transcription levels of these *spo0A* kinases, we observe a relative reduction in the expression of kinase *kinE* by 61% (Figure 4B). Looking at related phosphotransferases which inhibit Spo0A activation, we note relative fold-increases in transcription among *rapB*, *rapI*, and *rapJ* of 3.11, 2.90, and 3.29 respectively (Figure 4C).

**Figure 5:**
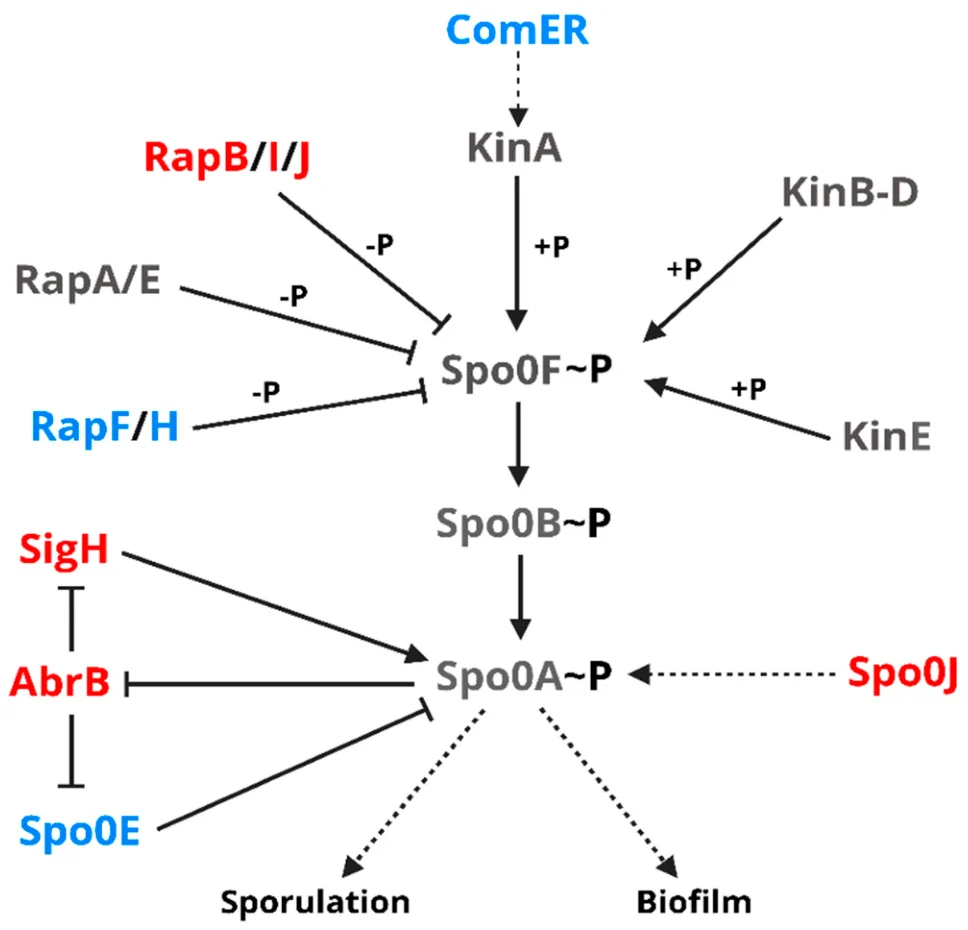
Schematic of the Spo0A regulatory pathway under the influence of HMAQ-7. Proteins colored red indicate upregulated transcriptional elements; those in blue indicate downregulated elements; and those in gray are not differentially expressed. Solid arrows indicate direct regulation, and dashed arrows indicate indirect regulation.

Taken together, the transcriptomics align with the idea that anti-colonization effects of HMAQ-7 in *B. subtilis* are the result of the compound augmenting components within the phosphorelay circuit (Figure 5). Furthermore, it does appear that the MoA of HMAQ-7 is consistent with an antagonistic parainduction mechanism, engendering global changes in gene expression and producing similar phenotypes as those previously observed in certain species exposed to AI-2 [71,72,73]. With that said, it is difficult to know whether the concomitant dysregulation in flagellar gene expression is directly attributable to the mechanism affecting sporulation or whether it is a separate downstream effect. Regardless, it is clear that HMAQ-7 subverts normative responses to nutrient deprivation during prolonged exposure and radically restructures *B. subtilis* physiology in ways that are highly relevant to future investigations.

### 2.4. Current Limitations and Future Directions

Our knowledge of the biological underpinnings of HMAQs has been quite limited until now, with some previous forays having provided useful, if not remote, findings. This study provides one of the first extensive insights into the physiological inductions of HMAQ’s in a model bacterial species. Although this is noteworthy, it is also necessary to acknowledge some of the work’s shortcomings as well as some future developments.

One of the major experimental limitations within the present study is the reliance upon biological duplicates for transcriptomics. While the use of duplicates provides statistically informative data, the low number of replicates does constrain the statistical robustness of our present analysis. This should be taken into consideration when evaluating genes displaying marginal statistical significance (0.01 < *p*adj < 0.05). With that said, both PCA and Pearson’s correlation analyses demonstrated strongly divergent responses in transcriptional expression between the available treated and control replicates (Figure S1). Furthermore, the fold changes observed amongst genes associated with sporulation/germination, biofilm/pellicle formation, and flagellar assembly all show associated *p*adj-values several orders of magnitude below the marginal significance threshold, with many of these genes also having log_2_FC values greater than 7 (FC ≥ 128-fold) (Table 1 and Table 2 and Tables S1–S3). All of this paired with our earlier experimental work provides strong assurance in our conclusion that HMAQ-7 exerts bona fide alterations in multiple virulence pathways, including in elements associated with the Spo0A phosphorelay system.

While we express robust confidence in our assessment of HMAQ-7 as a parainducer with noticeable, albeit unintuitive, modulatory effects, the available data do not provide a precise causal mechanism for how this is achieved. Although the relative transcriptional patterns among Spo0A phosphorelay genes are consistent with our hypothesis that HMAQ-7 disrupts signal transduction through the Spo0A phosphorelay in *B. subtilis*, transcriptional changes alone cannot establish this mechanism. This is due in large part to the non-linear association between transcriptional expression, translation, and enzymatic activity in cell culture [74,75]. Consequently, direct measurement of intracellular phosphorylation states in HMAQ-7-treated and untreated cells will be necessary to determine whether disruption of phosphotransduction contributes to the observed biological changes.

The current study also documents transcriptomic remodeling at only a single time point which limits the level of context we have for cellular adaptation. This is critical as this reduces our detection of physiologically relevant responses such as ROS production. ROS detection assays performed on the culture at 24 h did not show an elevation of ROS in treated cells (Figure S5). With that said, we cannot exclude the possibility of transient ROS accumulation and subsequent dissipation in the preceding 12 h. If indeed HMAQ-7 causes a change in intracellular phosphorylation, it would entail a decoupling of oxidative phosphorylation and a commensurate increase in ROS within the cell. This response would indeed be consistent with similar varieties of HMAQs which are known to achieve cellular dysfunction through oxidative stress. The implications of this hypothesis are interesting and warrant further investigation.

Beyond some of these limitations, the study does offer critical insights into *B. subtilis* ecology and soil species relationships more broadly. Transcriptomics identified relative upregulation of *luxS* transcription in treated cells, hinting at a potential connection between the signaling of HMAQs and AI-2. Previous reports have indicated that Spo0A negatively controls expression of *luxS* and, by extension, the synthesis of AI-2 [76]. If HMAQ-7 does in fact disrupt phosphorylation of spo0A directly, we might hypothesize that HMAQs mediate cross-species signaling through indirect methods. Although highly speculative, such a finding would suggest a more complex dimension to interspecies communication between *Burkholderia* and *Bacillus* than previously thought. This interactivity could, if demonstrated, be potentially interesting in studying these effects on other more distantly related and pathogenic varieties of Gram-positive species.

## 3. Materials and Methods

### 3.1. Bacterial Strains and Growth Conditions

*Bacillus subtilis* strain BS34a was cultured on either Todd-Hewitt yeast extract (THyex) medium or Tryptic Soy Agar (TSB) as mentioned previously [13,15]. *Burkholderia contaminans* MS14 was cultured on either NBY medium or in a modified M9 media containing L-asparagine (1 g/L L-asparagine).

### 3.2. Purification of HMAQ-7

Isolation of HMAQ-7 was conducted in Gu et al., 2011, with specific modifications [11]. The producing strain, *Burkholderia contaminans* MS14, was grown in 1 L of modified minimal M9 media with asparagine (1 g/L). The final pH of the medium was adjusted to 6.9. A 10% inoculum with an OD600 nm between 0.6 and 0.8 was made before incubating the culture at 28 °C for 3 to 4 days. Before drying, the cell-free supernatant (CFS) was passed through a 0.2 μm filter. The dried material was then rinsed with 200 mL of acetonitrile, which extracts HMAQ-7. The acetonitrile fraction containing HMAQ-7 was lyophilized and subsequently suspended in a sufficient volume of 80:20 acetonitrile:water (*v*/*v*) with 0.1% trifluoroacetic acid to re-solubilize the contents. This preparation was then subjected to reverse phase chromatography using a 5-micron C18 SinChrom ODS-BP (10 mm × 250 mm) preparative column employing a DuoFlow HPLC (Bio-Rad Laboratories, Hercules, CA, USA) at a mobile phase flow rate of 3 mL/min at room temperature. LC separations were performed using 10 mM Ammonium formate in water (MPA) and ACN (MPB) serving as the mobile phase (MP). The chromatography method consisted of a gradient elution program specified as follows: 0–5.0 min, 10% MPB; 5.0–32.0 min, 10–75% MPB; 32.0–37.0 min, 75% MPB; 37.0 min, 75–10% MPB; and 37.0–42.0 min, 10% MPB. The fraction of interest eluted at 75% MPB and was analyzed via MALDI-TOF for material validation. After confirming the product’s mass, the purified sample was lyophilized and weighed on an analytical scale (Adventurer Pro AV114C; Ohaus Corporation, Parsippany, NJ, USA). The powder would then be suspended in dimethyl sulfoxide (DMSO) for bioassays.

### 3.3. Standing Culture Preparation

Pellicle experiments were organized in accordance with methods discussed in Pisithkul et al., 2019, with specific modifications [77]. Briefly, a plate of an indicator species is inoculated into TSB containing 0.25% sucrose to an OD_600_ of between 0.2 and 0.3. After reaching the desired density, a 100-fold dilution is then performed into 10 mL of identical media containing either 10 μg/mL of HMAQ-7 or an equivalent volume of DMSO which acts as a vehicle. The inoculum of each tube is thoroughly vortexed before being decanted into sterile Petri dishes (VWR Polystyrene, 100 × 15 mm) and incubated at 37 °C for 24 h. After incubation, the contents of these plates would be pipetted into pre-weighted falcon tubes and centrifuged at 10,000 RPM for 5 min at 20 °C. The supernatants are subsequently decanted, and the pellets are resuspended in 1 mL of RNA shield (Zymo Chemicals, San Leandro, CA, USA) and stored on dry ice for transport. CFU/mL were determined with 10-fold serial dilutions of the tubes which were performed down to 10^−7^ and plated to ascertain cell density. Standard culture used for RNA-seq was n = 2 biological duplicates.

### 3.4. Preliminary Growth Curve Study

The growth curve study with *B. subtilis* was performed as described by Geng et al., 2018, with specific modifications [78]. Briefly, a fresh plate of *B. subtilis* 34A was incubated between 16 and 24 h at 37 °C. A tube containing 10 mL of fresh TSB broth + 0.25% sucrose was inoculated to a starting OD_600_ of 0.1 and incubated in a mechanical shaker. The tube remained incubated at 37 °C with a 240 rpm until reaching an OD_600_ of 0.5–0.6, at which point a 100-fold dilution is performed on the inoculum, resulting in a 2.3 × 10^5^ CFU/mL cell density. HMAQ-7 material was solubilized into a 10 mg/mL stock solution using 100% DMSO. The volume of experimental tubes was adjusted such that the final working concentration would be 10 μg/mL. An equivalent volume of DMSO (1% vol) would be used as vehicle controls absent the drug. Treatments and vehicle tubes were made in duplicate. Both sets of tubes would be incubated at 37 °C and 240 rpm. A 100 μL aliquot would be taken from each tube at 0, 2, 4, 8, 12, and 24 h time points. Readouts between 0.000 and 0.900 would be used for charting growth, with readouts beyond this range undergoing 2-fold dilution. Bars represent technical duplicates of experiments (n = 2).

### 3.5. RNA-Seq Library Preparation

Two biological replicates of both treated and non-treated *B. subtilis* standing culture samples were sent to Zymo Research for Total RNA-Seq Service (Zymo Chemicals, San Leandro, CA, USA). Libraries were built from total RNA samples, with a full extraction being performed on each replicate. No samples were excluded from processing. Library preparation was performed using the Zymo-Seq RiboFree Total RNA Library Prep Kit (Cat # R3000) (Zymo Chemicals, San Leandro, CA, USA) according to the manufacturer’s instruction manual v1.3.1. Briefly, RNA was reverse-transcribed into cDNA, which was followed by ribosomal RNA depletion. Afterwards, the partial P7 adapter sequence was ligated to the 3′ end of cDNAs, followed by subsequent second-strand synthesis and partial P5 adapter ligation to the 5′ end of the double-stranded DNAs. Libraries were amplified to integrate full-length adapters under the following conditions: initial denaturation at 95 °C for 10 min; 10–16 cycles of denaturation at 95 °C for 30 s, annealing at 60 °C for 30 s, and extension at 72 °C for 60 s; and final extension at 72 °C for 7 min. Successful library construction was validated with Agilent’s D1000 ScreenTape Assay on TapeStation (Agilent Technologies, Santa Clara, CA, USA). RNA-Seq libraries were sequenced on an Illumina NovaSeq to a sequencing depth of at least 30 million read pairs (150 bp paired-end sequencing) per sample (Illumina, San Diego, CA, USA).

### 3.6. RNA-Seq Data Bioinformatics Analysis

The Zymo Research RNA-Seq pipeline was originally adapted from nf-core/rnaseq pipeline v1.4.2 (https://github.com/nf-core/rnaseq) [79]. The pipelines were built using Nextflow (https://www.nextflow.io/) [80]. Briefly, quality control of raw reads was carried out using FastQC v0.11.9 (http://www.bioinformatics.babraham.ac.uk/projects/fastqc). Adapter and low-quality sequences were trimmed from raw reads using Trim Galore! v0.6.6 (https://www.bioinformatics.babraham.ac.uk/projects/trim_galore). Trimmed reads were aligned to the reference genome using STAR v2.6.1d (https://github.com/alexdobin/STAR) [81]. BAM file filtering and indexing were carried out using SAMtools v1.9 (https://github.com/samtools/samtools) [82]. RNAseq library quality control was implemented using RSeQC v4.0.0 (http://rseqc.sourceforge.net/) [83] and QualiMap v2.2.2-dev (http://qualimap.conesalab.org/) [84]. Duplicate reads were marked using Picard tools v2.23.9 (http://broadinstitute.github.io/picard/) [85]. Library complexity was estimated using Preseq v2.0.3 (https://github.com/smithlabcode/preseq) [86]. Duplication rate quality control was performed using dupRadar v1.18.0 (https://bioconductor.org/packages/dupRadar/) [87]. Reads overlapping with exons were assigned to genes using featureCounts v2.0.1 (http://bioinf.wehi.edu.au/featureCounts/) [88]. Classification of rRNA genes/exons and their reads were based on annotations and RepeatMasker rRNA tracks from UCSC genome browser when applicable. Differential gene expression analysis was completed Ver 3.0 using DESeq2 v1.28.0 (https://bioconductor.org/packages/DESeq2/) [89]. Functional enrichment analysis was achieved using g:Profiler python API v1.0.0 (https://biit.cs.ut.ee/gprofiler/gost) [90]. Quality control and analysis results plots were visualized using MultiQC v1.9 (https://github.com/ewels/MultiQC) [91]. All are accessed on 9 September 2026.

As part of the DESeq2 design, we excluded all genes from the “FeatureCounts” counts matrix that had zero counts in all samples, then applied DESeq2 rlog counts normalization with blind set to false. The DESeq2 design formula used in the comparison involved only one variable/group, which represented the treated and control conditions. DESeq2 employed the Benjamini–Hochberg (BH) correction to generate *p*adj values. RNA counts were modeled with a negative binomial distribution and employed the Wald test in all differential expression calculations.

### 3.7. MA Plotting and Enrichment Analyses

An MA plot was generated using the normalized base mean transcripts. A DEG was defined based on the following thresholds: (*p*adj < 0.05; |Log2FC| > 1.5). GO term, KEGG, and pathview enrichment analyses were used to assess the transcriptomics profile of BS34A data. All omics-based analyses were subject to Benjamin–Hochberg correction to minimize the False Discovery Rate. Due to low biological replicates, an exploratory *p*-value was used for pathview analysis to capture borderline genes of biological interest. As a result of the limited curation of the BS34A strain, detected genes were mapped to their corresponding orthologs in the better-curated *B. subtilis* 168 strain using the smart table tool in BioCyc. *B. subtilis* 168 would serve as the reference genome for all enrichment analyses.

### 3.8. ROS Detection Experiment

A ROS-ID^®^ Total ROS/Superoxide detection kit from Enzo Biochemistry was used according to the instructions of the manufacturer. For experiments with *B. subtilis*, cells were initially cultured and prepped as described in Section 3.3. 10 µg/mL of HMAQ-7 was added to each tube or an equivalent volume of DMSO before mixing and storage in sterile Petri dishes. Plates were incubated for 24 h, after which the contents were collected via a serological pipette and transferred into Falcon tubes. In total, 2 µL of the ROS detection reagent would be added to both sets of tubes at a final concentration of 1 uM, vortexed, and then stored for 30 min at 37 °C. After 30 min readings would be made as described previously.

All measurements were taken on a Biorad Versafluor fluorometer set at medium gain, 1000 acquisition range, and blanked with Thyex media. In total, 1 mM hydrogen peroxide was used as a detection control. A 490/520 Excitation/Detection Filter was employed for these experiments. All bar graphs represent technical duplicates of experiments (n = 2).

### 3.9. Statistical Analysis

Statistically significant differences between treatment and solvent controls in terms of CFU/mL and OD_600_ were determined via a two-sample t-test. A statistically significant difference was defined as one with a minimum measured *p* < 0.05 after appropriate Bonferroni correction. All statistical analyses were performed in Graphpad Prism ver. 9.5.0.

## 4. Conclusions

This transcriptomics-based study into HMAQ-7 activity constitutes one of the first investigations into the specific MoA of an HMAQ compound. RNA-seq analysis has provided evidence for HMAQ-7 achieving its antimicrobial effects through the transcriptional attenuation of components involved in intracellular phospho-transduction, including the Spo0A phosphorelay circuit (Figure 5). These might explain the clear reductions in biofilm formation and sporulation phenotypes previously seen in treated *B. subtilis* [15]. Furthermore, transcriptomics and bioinformatics identify an apparent dysregulation in the expression of flagellar assembly, which likely contributes to cellular starvation experienced by the cell during late stationary phase.

## Figures and Tables

**Table 1:** Top 25 downregulated genes in *B. subtilis*-treated cultures following exposure to HMAQ-7 during comparative transcriptomic analysis.

Gene Name	Function	|Log_2_FC|	Adj *p*-Value
*yhzE1 (sscA)*	Spore assembly and germination protein	14.5	6.57796 × 10^−20^
*cotF*	Spore coat protein	12.4	1.37096 × 10^−36^
*cmpA*	Cortex morphogenic protein A; represses cortex assembly until the successful initiation of spore coat assembly	12.2	9.67629 × 10^−14^
*ykzV*	Unknown (Associated with Sporulation)	12.1	1.7825 × 10^−119^
*cotNE*	Inner spore coat protein	11.6	8.2342 × 10^−104^
*VV28_RS09090*	Unknown	11.5	3.81043 × 10^−50^
*VV28_RS19655*	Unknown	11.4	5.9298 × 10^−178^
*yhzE2* (*sscB*)	Spore and germination protein	11.2	7.22395 × 10^−12^
*cotY*	Main structural component of the spore crust; necessary for the assembly of the spore crust, the outermost layer of the spore coat	11.2	2.7225 × 10^−158^
*yqfT*	Unknown (Associated with Sporulation)	11.0	2.84746 × 10^−81^
*yppG*	Spore coat protein	10.9	1.22676 × 10^−48^
*spoVIF*	Required for spore coat assembly and resistance	10.9	8.3287 × 10^−99^
*VV28_RS05475*	Unknown	10.8	2.65183 × 10^−39^
*cotZ*	Spore crust anchor protein; necessary for the assembly of the spore crust, the outermost layer of the spore coat	10.8	2.297 × 10^−99^
*glnH*	Glutamine ABC transporter (binding protein)	10.8	4.97431 × 10^−58^
*ydgB*	Unknown	10.6	6.98086 × 10^−26^
*ytcB*	Putative UDP-glucose epimerase (expressed late during sporulation in mother cell)	10.6	6.89041 × 10^−70^
*gerPF*	Spore germination protein; facilitates access of nutrient germinants to their cognate germinant receptors in spores’ inner membrane	10.6	7.81655 × 10^−67^
*ytzH*	Unknown (Expressed during sporulation)	10.2	1.15147 × 10^−10^
*cotW*	Spore crust protein (insoluble fraction); involved in the formation of the spore crust	10.2	2.2383 × 10^−119^
*yitB*	Phospho-adenylylsulfate sulfotransferase	10.2	2.72395 × 10^−52^
*yjcZ*	Unknown (Associated with sporulation)	10.1	2.5711 × 10^−72^
*ytcA*	Similar to UDP-sugar dehydrogenase	10.1	2.39958 × 10^−84^
*cotX*	Spore crust protein (insoluble fraction); necessary for the assembly of the spore crust, the outermost layer of the spore coat	10.0	2.8129 × 10^−138^
*cotV*	Spore crust protein (insoluble fraction); involved in the formation of the spore crust	10.0	2.9889 × 10^−138^

**Table 2:** Top 25 upregulated genes in *B. subtilis* treated cultures following exposure to HMAQ-7 during comparative transcriptomic analysis.

Gene Name	Function	|Log_2_FC|	Adj *p*-Value
*pchE*	Pyochelin synthetase (siderophore)	7.9	5.62941 × 10^−55^
*cypX*	cytochrome P450, cyclo-l-leucyl-l-leucyl dipeptide oxidase	7.7	8.35828 × 10^−60^
*pchR*	transcriptional repressor (MarR family); controls the expression of genes involved in pulcherriminic acid biosynthesis	7.6	1.10441 × 10^−52^
*gmuA*	glucomannan-specific permease of the phosphotransferase system; EIIA of the PTS	7.6	6.47966 × 10^−74^
*gmuD*	phospho-beta-mannosidase	7.4	2.05897 × 10^−73^
*pchC*	Thioesterase (Optimizes Biosynthesis of Pyochelin)	7.4	2.40079 × 10^−57^
*celB*	lichenan-specific permease of the phosphotransferase system, EIIC of the PTS	7.3	5.36079 × 10^−71^
*gmuB*	glucomannan-specific permease of the phosphotransferase system, EIIB of the PTS	7.1	3.87635 × 10^−63^
*manA (yjdE)*	mannose-6-phosphate isomerase, required for proper cell wall synthesis (mutants lose rod shape)	6.6	6.99917 × 10^−59^
*gmuR*	transcriptional repressor (GntR family) of the gmuB-gmuA-gmuC-gmuD-gmuR-gmuE-gmuF-gmuG operon	6.6	4.97431 × 10^−58^
*gmuE*	mannose kinase	6.5	2.27548 × 10^−59^
*czcD*	cation exporter (antiporter); involved in the export of zinc in exchange for extracellular K+ and H+	6.1	4.25716 × 10^−44^
*gmuG*	beta-1,4-mannanase	5.9	9.99789 × 10^−53^
*treP*	trehalose permease of the phosphotransferase system; EIIBC of the PTS	5.6	4.87397 × 10^−46^
*ybeC (aimA)*	general amino acid importer; low affinity symporter for glutamate/H+, serine/H+, asparagine/H+, glycine[metabolite|]/H+, diaminopropionic acid/H+, alanine/H+, and beta-alanine/H+	5.6	7.19505 × 10^−36^
*copO*	metal (copper) efflux transporter	5.6	7.64361 × 10^−40^
*amtB*	ammonium transporter; required at low ammonium concentration	5.5	4.15004 × 10^−32^
*treC (treA)*	phospho-alpha-glucosidase	5.4	2.19579 × 10^−35^
*rbsB*	ribose ABC transporter (binding protein)	5.1	6.61476 × 10^−31^
*msmR (melR)*	transcriptional regulator of the melR-melE-melD-melC-melA operon	5.1	1.01571 × 10^−23^
*rbsC*	ribose ABC transporter (permease)	5.1	2.91855 × 10^−32^
*sdhB*	succinate dehydrogenase	5.0	1.10992 × 10^−23^
*cimH*	transporter for citrate (proton symport)	5.0	1.14098 × 10^−30^
*cadA*	Cadmium-transporting ATPase, resistance to cadmium	4.9	1.3597 × 10^−34^
*bdhA (ydjL)*	acetoine/butanediol dehydrogenase	4.9	6.63549 × 10^−29^

**Table 3:** List of genes involved in the Spo0A phosphorelay.

Gene Name	log2FC	*p*adj	Relative Transcription *
*comER*	−6.789799858	3.12783 × 10^−53^	0.009 ± 0.000
*spo0A*	−0.805853708	0.804073008	0.572 ± 0.082
*spo0B*	−0.290363342	1	0.881 ± 0.401
*spo0E*	−1.566555243	0.036105837	0.357 ± 0.102
*spo0F*	−0.632207452	1	0.650 ± 0.127
*spo0J*	1.79625086	0.003607347	3.604 ± 1.462
*kinA*	−0.089142854	1	0.940 ± 0.000
*kinB*	0.416301095	1	1.257 ± 0.430
*kinC*	0.785421498	0.868700963	1.722 ± 0.390
*kinD*	1.257651556	0.141049911	2.384 ± 0.077
*kinE*	−1.37884717	0.061589895	0.390 ± 0.106
*rapA*	0.059891589	1	1.112 ± 0.322
*rapB*	1.640453482	0.008435424	3.111 ± 0.398
*rapE*	0.844007532	0.893652685	1.737 ± 0.930
*rapF*	−3.538468751	3.19951 × 10^−11^	0.084 ± 0.037
*rapH*	−1.900906358	0.000592772	0.271 ± 0.054
*rapI*	1.49720248	0.036105837	2.904 ± 0.750
*rapJ*	1.671685903	0.008095299	3.294 ± 1.043
*sigH*	2.118787383	4.60775 × 10^−5^	4.339 ± 0.602
*abrB*	1.901515014	0.002127456	3.953 ± 1.978

* Values corresponding to those calculated and displayed in Figure 4A.

## Data Availability

The original contributions presented in this study are included in the article/Supplementary Materials. Further inquiries can be directed to the corresponding author. The RNA-seq data have been deposited in the NCBI Sequence Read Archive (SRA). The BioProject accession number is PRJNA1236414.

## Supplementary Materials

The following supporting information can be downloaded at https://www.mdpi.com/article/10.3390/molecules31183226/s1. Table S1: Genes involved in Flagellar Assembly with Corresponding Directional Log_2_ Fold Changes. Table S2: Genes involved in Chemotaxis with Corresponding Directional Log_2_ Fold Changes. Table S3: Genes involved in Pellicle/Biofilm Biosynthesis with Corresponding Direction Log_2_ Fold Changes. Figure S1: PCA and Pearson Correlation of B. subtilis Samples. Figure S2: KEGG Analysis. Figure S3: Flagellar assembly in *B. subtilis.* Figure S4: Two-component systems in *B. subtilis.* Figure S5: Induction of ROS in *B. subtilis*.

## Abbreviations

HMAQ	4-hydroxy-3-methyl-2-alkylquinoline
HMAQ-7	4-hydroxy-3-methyl-2-alkylquinoline-C7
MoA	Mode of Action
QS	Quorum Sensing
HAQ	2-heptyl-alkylquinoline
MEC	Minimum Effective Concentration
PCA	Principal Correlation Analysis
AI-2	Autoinducer-2
DEG	Differentially Expressed Gene
BP	Biological Process
CC	Cellular Component
KEGG	Kyoto Encyclopedia of Genes and Genomes
TCS	Two-Component System
ROS	Reactive Oxygen Species
THyex	Todd-Hewitt Yeast Extract
TSB	Tryptic Soy Broth
CFS	Cell Free Supernatant
DMSO	Dimethyl Sulfoxide
