# Supplementary material for: 4-Hydroxy-3-Methyl-2-Alkenylquinoline Disrupts Temporospatial Control of Flagellar Assembly and Induces Transcriptomic Remodeling in Bacillus subtilis Consistent with a Short-Circuiting of the Spo0A Phosphorelay
Source: Molecules. 2026 Sep 12;31(18):3226. doi: 10.3390/molecules31183226 (PMC13609334; doi:10.3390/molecules31183226)
Supplement: Supplementary file 1 [file molecules-31-03226-s001.zip › molecules-4518570-supplementary.pdf]

4-Hydroxy-3-Methyl-2-Alkenylquinoline Disrupts Temporospatial Control of Flagellar Assembly and Induces Transcriptomic Remodeling in *Bacillus subtilis* Consistent with a Short-Circuiting of the Spo0A Phosphorelay

McKinley D. Williams [1,2] and Leif Smith [1,3,*]

[1] Department of Biology, Texas A&M University, College Station, TX 77843, USA; mcdwilliams@pvamu.edu

[2] Department of Biology, Prairie View A&M University, Prairie View, TX 77446, USA

[3] Antimicrobial Division, Sano Chemicals Inc., Bryan, TX 77808, USA

[*] Correspondence: jsmith@bio.tamu.edu

Tables

Table S1: Genes involved in Flagellar Assembly with Corresponding Directional Log_2 Fold Changes

Table S2: Genes involved in Chemotaxis with Corresponding Directional Log_2 Fold Changes

Table S3: Genes involved in Pellicle/Biofilm Biosynthesis with Corresponding Direction Log_2 Fold Changes

Figures

Figure S1: PCA and Pearson Correlation of B. subtilis Samples

Figure S2: KEGG Analysis.

Figure S3: Flagellar assembly in *B. subtilis.*

Figure S4: Two-component systems in *B. subtilis.*

Figure S5: Induction of ROS in *B. subtilis.*

Table S1: Genes involved in Flagellar Assembly with Corresponding Directional Log_2 Fold Changes

Gene Name	Log_2FC	P_{adj}
flgB	1.982845659	0.000315138
flgC	2.404207714	7.7262E-07
flgD	2.990061146	4.26914E-09
flgG	2.777652087	1.68713E-07
flgK	3.769171831	6.86873E-19
flgL	3.655141117	5.60284E-16
flgM	3.366077778	1.84773E-13
flgN	3.021674246	1.03179E-10
flhA	2.873740099	4.83738E-09
flhB	3.179254769	8.57329E-10
flhF	3.15546347	7.78558E-10
flhG	2.989572276	2.54744E-09
flhO	1.763503093	0.003584667
flhP	1.875815401	0.002768506
fliD	2.002722886	0.00034327
fliE	3.436857962	1.54012E-14
fliF	3.05292211	8.31758E-12
fliG	3.271738892	2.29135E-13
fliH	3.409249755	1.74093E-12
fliI	3.211380931	3.39483E-10
fliJ	3.545570544	9.13324E-12
fliK	3.270819256	4.06443E-11
fliL	3.164466435	2.4522E-10
fliM	3.102751926	7.1986E-10
fliP	3.596219714	1.9283E-14
fliQ	3.8567602	2.92119E-13
fliR	3.142199214	1.33274E-10
fliS	2.094608448	8.63808E-05
fliT	2.071405364	0.000127514
fliW	0.950473608	0.592823436
fliY	2.619529713	2.65013E-07
fliZ	3.646004338	6.71111E-14
hag	2.591484674	8.63914E-07

Table S2: Genes involved in Chemotaxis with Corresponding Log_2 Fold Changes

Gene Name	Log_2FC	P_{adj}
cheA	1.8922143	0.001967417
cheB	2.879506794	1.34366E-08
cheC	1.641239896	0.015478546
cheD	2.266132176	3.50378E-05
cheR	1.079215606	0.369229924
cheV	1.259422094	0.121799342
cheW	1.58071401	0.026930101
cheY	2.855292916	1.70186E-08
motA	3.055265307	1.05933E-09
motB	2.909372367	2.12833E-08
motP	1.635764883	0.068269348
motS	-1.415471037	0.247068394

Table S3: Genes involved in Pellicle/Biofilm Biosynthesis

Gene	Log_2FC	P_{adj}
epsA	-6.260031066	1.75248E-33
epsB	-5.539592651	1.14947E-25
epsC	-0.16716238	1
epsD	-2.830317544	4.68999E-07
epsE	-3.023276739	2.13184E-07
epsF	-2.943579935	4.83775E-08
epsG	-3.252281201	1.89643E-09
epsH	-3.210046772	1.29554E-07
epsI	-4.008552574	1.20804E-15
epsJ	0.168319956	1
epsK	-0.135224868	1
epsL	-3.215285224	2.44426E-08
epsM	-3.523544141	6.83507E-11
epsN	-4.442000709	1.57454E-19
epsO	-4.708149179	4.00433E-18
sipW	-9.140997883	3.75404E-54
tapA	-8.09438773	5.90121E-48
tasA	-9.771538209	2.5686E-117

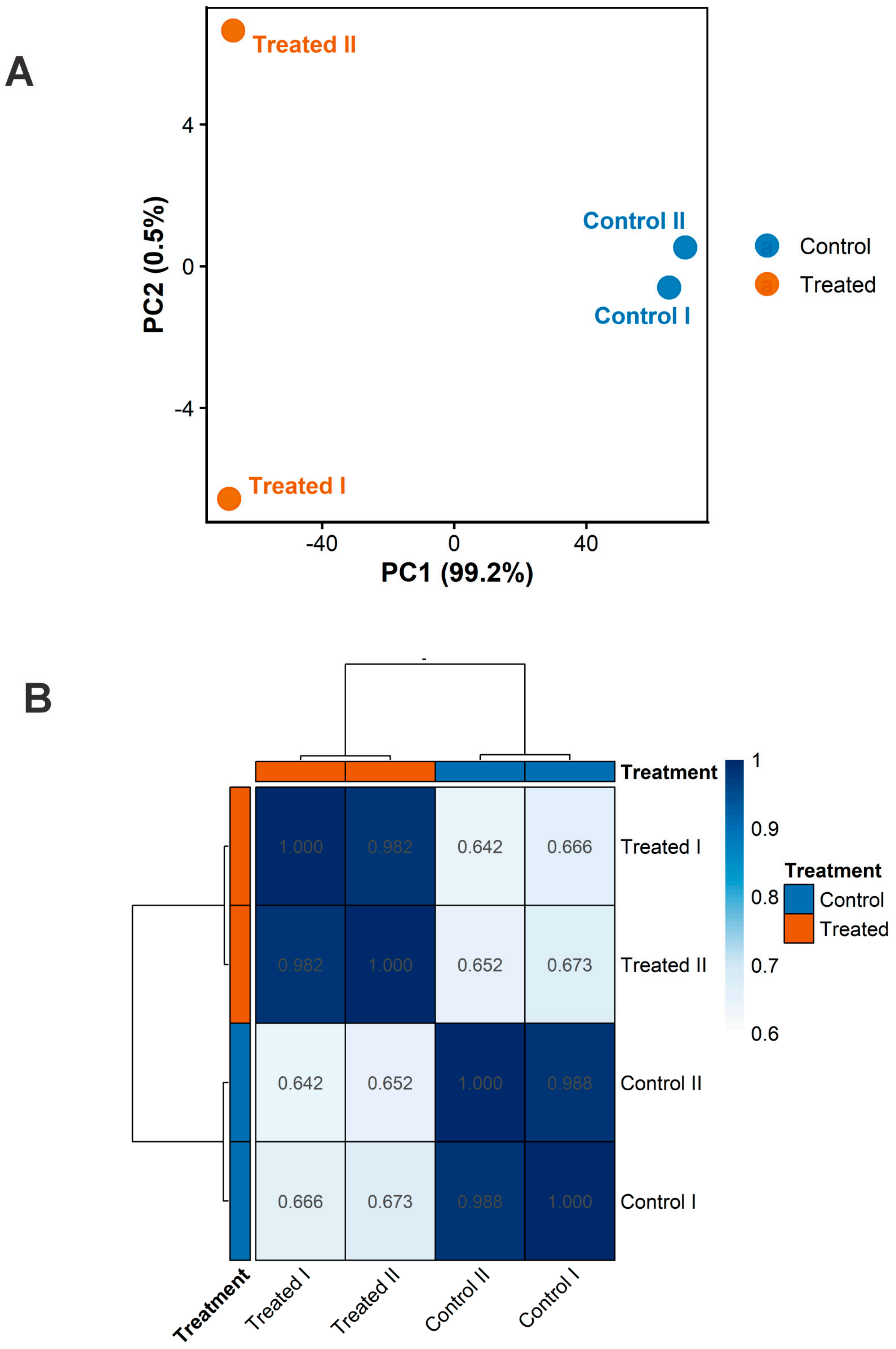

Figure S1: Exposure to HMAQ-7 induces a distinct transcriptional adaptation in treated cells when compared to control. A.) PCA plot of treated and control B. subtilis cultures. B.) Corresponding Pearson Correlation HeatMap

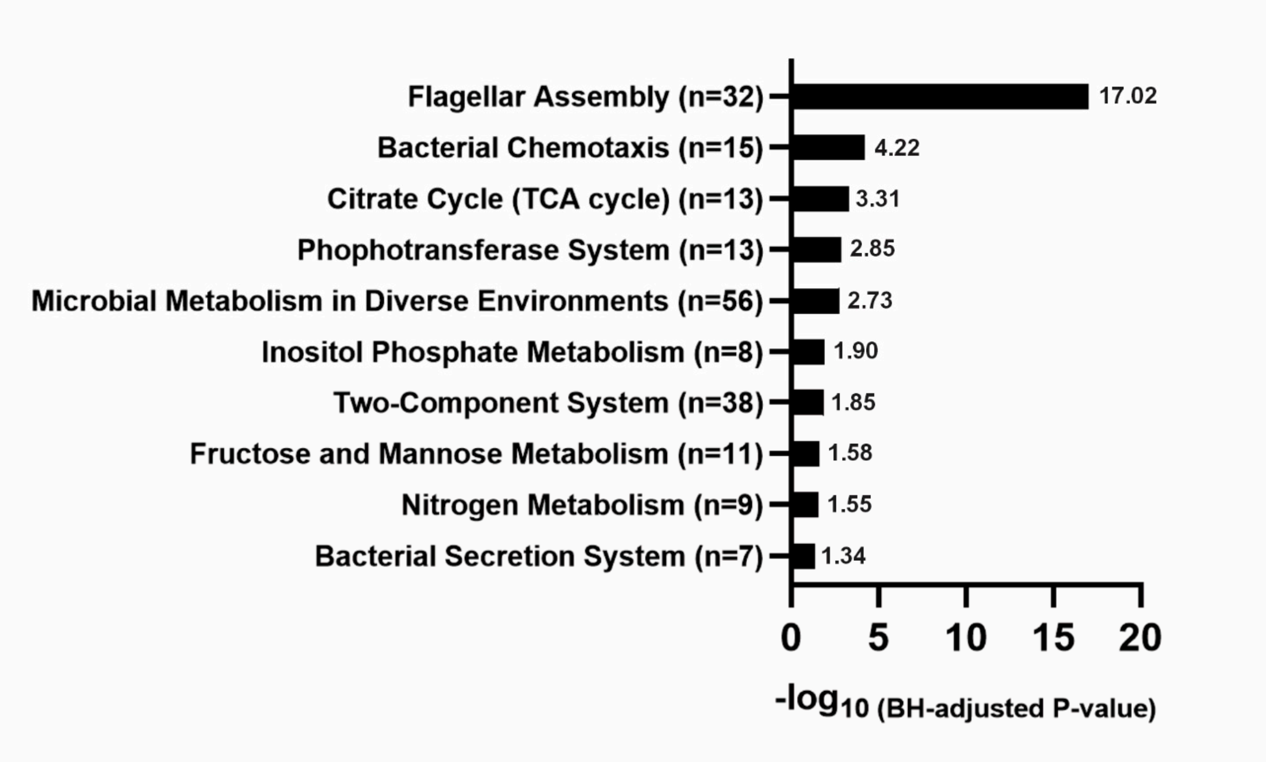

Figure S2: KEGG Enrichment. HMAQ-7 promotes several pathways involved in motility, cellular metabolism, and flagellar assembly in *B. subtilis*. KEGG analysis identifies a number of pathways, shown in order of enrichment, associated with significant ($p<0.05$) upregulation. The “n” next to each pathway denotes the number of genes therein.

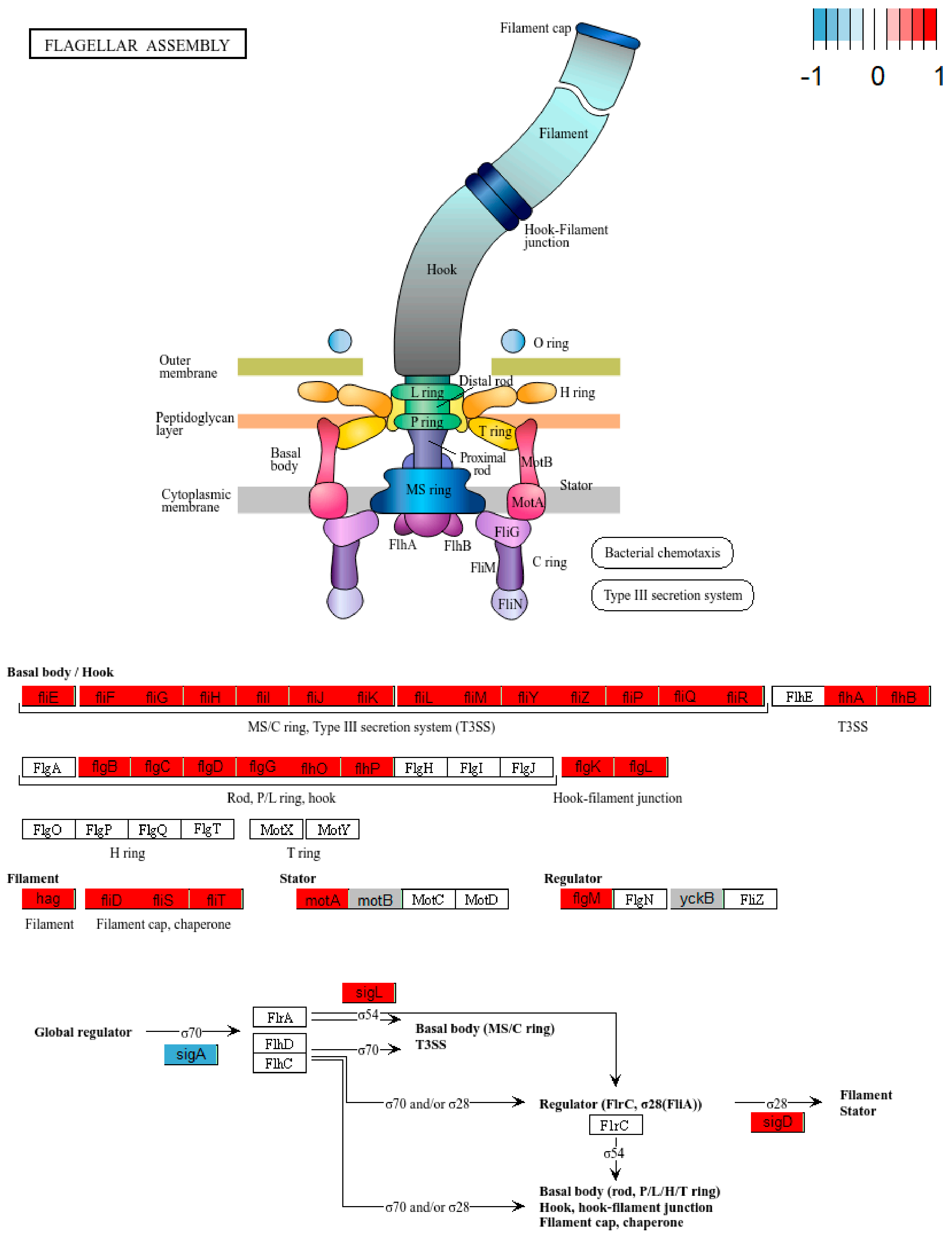

Figure S3: Flagellar assembly in *B. subtilis*. HMAQ-7 promotes upregulation of genes involved in flagellar assembly in *B. subtilis,* particularly in the basal body and hook. *sigL* and *sigD* expression are upregulated despite the upstream housekeeping regulator, *sigA*, being downregulated. Data on KEGG graph and rendered by Pathview.

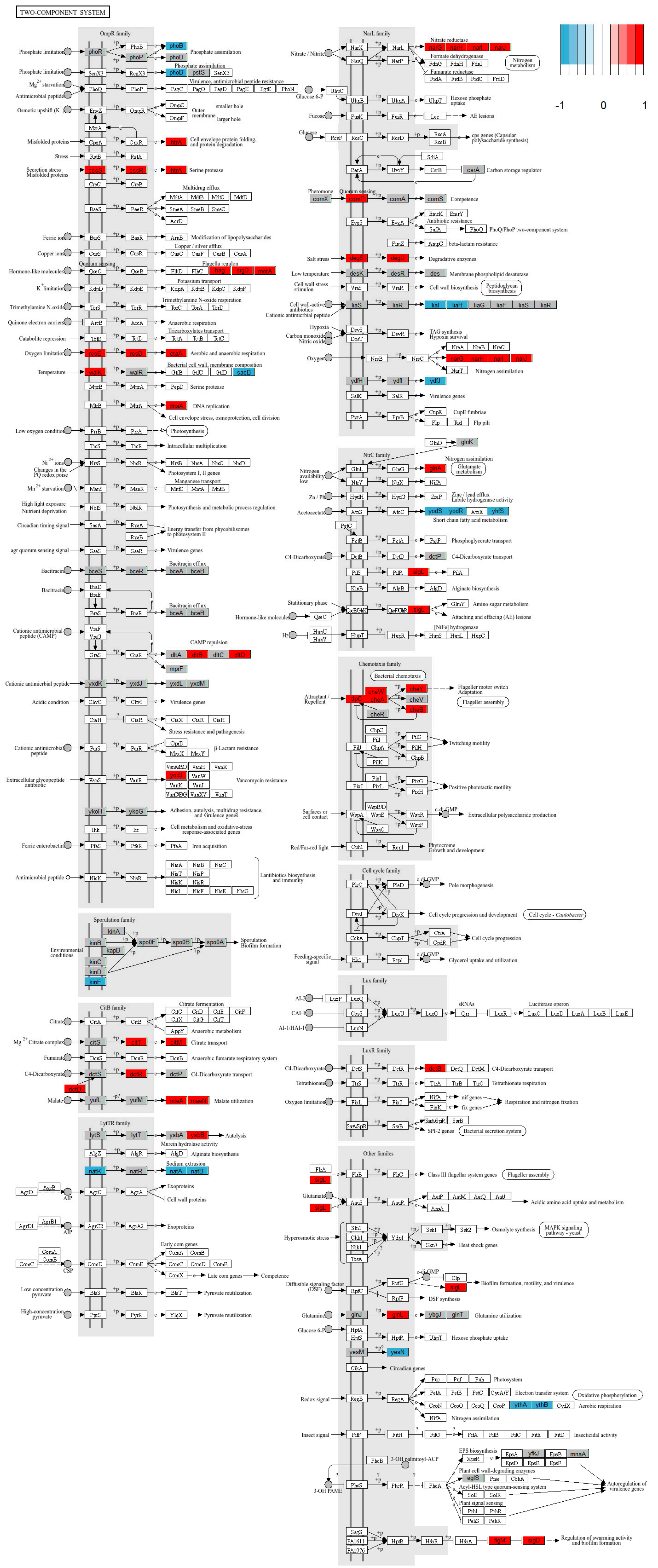

Figure S4: Two-component systems in *B. subtilis*. HMAQ-7 modulates several genes associated with two-component systems in *B. subtilis* including systems tied to sporulation/biofilm, phosphate assimilation, competence, and aerobic respiration. Data on KEGG graph and rendered by Pathview.

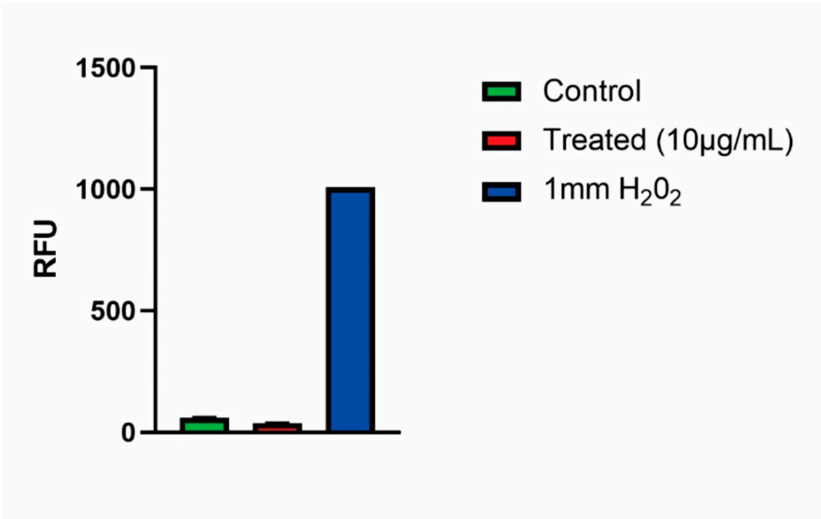

Figure S5: Induction of ROS in *B. subtilis.* Unlike similar compounds, HMAQ-7 does not induce ROS production in *B. subtilis.* 24hrs incubation with HMAQ-7 results in significantly lower level of ROS compared to control.
